# Draft Genome Sequences of Antibiotic-Resistant *Serratia* sp. Strains Isolated from Raw Sewage and Sediment Sludge in Georgia, USA

**DOI:** 10.1128/mra.00965-22

**Published:** 2022-11-16

**Authors:** Jouman Hassan, Malak A. Esseili, David Mann, Marwan Osman, Shaoting Li, Xiangyu Deng, Issmat I. Kassem

**Affiliations:** a Center for Food Safety, Department of Food Science and Technology, University of Georgia, Griffin, Georgia, USA; b Department of Public and Ecosystem Health, College of Veterinary Medicine, Cornell University, Ithaca, New York, USA; c Cornell Atkinson Center for Sustainability, Cornell University, Ithaca, New York, USA; University of Maryland School of Medicine

## Abstract

Sewage has been established as a prime matrix for monitoring the emergence and dissemination of etiologic agents and antibiotic resistance determinants in a population. Here, we report the draft genomes and the acquired resistance genes of 11 antibiotic-resistant *Serratia* sp. isolates that were detected in samples from wastewater treatment plants.

## ANNOUNCEMENT

We noted the occurrence of multidrug-resistant (MDR) isolates that exhibited high levels of resistance to colistin in sewage samples collected in Georgia, USA (1). However, those isolates lacked an *mcr* amplicon (*mcr-1* to *mcr-9*). To identify the strains and their antibiotic resistance determinants, we selected 11 isolates for whole-genome sequencing.

The strains were isolated from composite influent sewage and sediment sludge samples (1 L) from wastewater treatment plants. An aliquot from each sample (100 μL) was spread onto RAPID'*E.coli*2 agar plates (Bio-Rad, USA) supplemented with 4 μg/mL colistin (Sigma-Aldrich, USA) (1–4). The plates were incubated at 37°C under aerobic conditions for 24 h, and colonies showing a deep purple color and white circumference were selected.

After purification on RAPID'*E.coli*2 agar as described earlier, colonies were transferred with inoculation loops and suspended in the buffer supplied in the QIAamp DNA minikit (Qiagen, USA) to isolate genomic DNA as described in the manufacturer’s protocols. The DNA was quantified using the Qubit double-stranded DNA (dsDNA) broad-range (BR) assay kit (Invitrogen, USA) (5, 6). The Nextera XT DNA library preparation kit and the Qubit dsDNA high-sensitivity (HS) assay kit (Invitrogen) were used to prepare and determine the concentrations of the sample libraries, respectively (7). The libraries were diluted, denatured according to Illumina protocol A (8), and loaded into the MiSeq reagent cartridge (MiSeq reagent kit v2, 300 cycles) (7). Sequencing was performed using the paired-end sequencing strategy (2 × 250 bp) with a MiSeq sequencer (Illumina, USA). Low-quality reads were removed with Trimmomatic v0.36 (9). The leading 3 nucleotides and the trailing 3 nucleotides were removed from the reads, and a 4-nucleotide sliding window was used to also remove nucleotides from the 3′ ends when the average Phred score dropped below 20. Reads shorter than 75 bp were discarded. Draft genome sequences were assembled from trimmed and filtered reads using the –careful option in SKESA v2.4.0 (10). Contigs shorter than 200 bp were discarded, and the quality of the draft genome was evaluated with QUAST v4.5 (11). The assemblies were annotated using PGAP v5.2 (12). The identity of the isolates was determined using the ribosomal multilocus sequence typing (rMLST) database (13). Acquired antibiotic resistance genes and the plasmid types were identified using the ResFinder v4.1 and PlasmidFinder v2.1 databases, respectively (14, 15). Default parameters were used for all software unless otherwise specified.

Analysis using rMLST showed that the isolates matched 100% with *Serratia marcescens* (16). However, the average nucleotide identity data (NCBI GenBank) showed that the best-matching type strain assembly (GCA_008364245.1) was Serratia nevei, a novel species described in 2020 (17). *Serratia* spp. are intrinsically resistant to ampicillin and amoxicillin (with or without β-lactamase inhibitors), first-generation cephalosporins, macrolides, and colistin, which explained the high colistin resistance exhibited by the isolates, as well as their resistance to other antibiotics (Table 1). The isolates carried three antibiotic resistance genes, namely, *aac(6′)-Ic*, *bla*_SRT-2_, and *tet*(41), and the IncFII(Yp) plasmid.

**TABLE 1:** Genome properties and antibiotic resistance profiles of *Serratia* sp. strains isolated from sewage collected from wastewater treatment plants in Georgia, USA

Source, sample type, and sample identification code^*a*^	Genome size (bp)	No. of contigs	*N*_50_ (bp)	*L*_50_	Genome coverage (×)	GC content (%)	Colistin MIC (μg/mL)^*b*^	Antibiotic resistance phenotype^*c*^	Acquired antibiotic resistance genes detected by ResFinder v4.1	Plasmid detected by PlasmidFinder v2.1	SRA accession no.	GenBank accession no.	GenBank assembly accession no.
Wastewater treatment plant 1													
Influent													
B1	5,320,010	432	26,787	60	85.14	57.9	<640	R: PEN, AMP, AMC, ERY, TET; S: FEP, CTX, CFM, DOR, IPM, MEM, GEN, KAN, STR, CIP, NOR, SXT, CHL	*aac(6′)-Ic*, *bla*_SRT-2_, *tet*(41)	IncFII(Yp)	SRX11631614	JAIHNA000000000.1	GCA_019732695.1
Sediment sludge													
B2	5,340,812	331	32,968	49	79.18	59.1	<640	R: PEN, AMP, AMC, ERY, TET; S: FEP, CTX, CFM, DOR, IPM, MEM, GEN, KAN, STR, CIP, NOR, SXT, CHL	*aac(6′)-Ic*, *bla*_SRT-2_, *tet*(41)	IncFII(Yp)	SRX11631613	JAIHNB000000000.1	GCA_019732665.1
B3	5,364,286	288	40,329	38	80.21	59.7	<640	R: PEN, AMP, AMC, ERY, TET; S: FEP, CTX, CFM, DOR, IPM, MEM, GEN, KAN, STR, CIP, NOR, SXT, CHL	*aac(6′)-Ic*, *bla*_SRT-2_, *tet*(41)	IncFII(Yp)	SRX11631612	JAIHNC000000000.1	GCA_019732635.1
A2	5,350,626	294	41,295	41	84.02	58.2	<640	R: PEN, AMP, AMC, ERY, TET; S: FEP, CTX, CFM, DOR, IPM, MEM, GEN, KAN, STR, CIP, NOR, SXT, CHL	*aac(6′)-Ic*, *bla*_SRT-2_, *tet*(41)	IncFII(Yp)	SRX11631625	JAIHML000000000.1	GCA_019733195.1
A4	5,376,369	212	66,460	27	89.32	59.2	<640	R: PEN, AMP, AMC, ERY, TET; S: FEP, CTX, CFM, DOR, IPM, MEM, GEN, KAN, STR, CIP, NOR, SXT, CHL	*aac(6′)-Ic*, *bla*_SRT-2_, *tet*(41)	IncFII(Yp)	SRX11631619	JAIHMM000000000.1	GCA_019733165.1
A10	5,360,467	280	50,770	35	81.14	59	<640	R: PEN, AMP, AMC, ERY, TET; S: FEP, CTX, CFM, DOR, IPM, MEM, GEN, KAN, STR, CIP, NOR, SXT, CHL	*aac(6′)-Ic*, *bla*_SRT-2_, *tet*(41)	IncFII(Yp)	SRX11631626	JAIHMS000000000.1	GCA_019733075.1
Wastewater treatment plant 2													
Sediment sludge													
A9	5,379,746	307	39,603	43	100.2	58.9	<640	R: PEN, AMP, AMC, ERY, TET; S: FEP, CTX, CFM, DOR, IPM, MEM, GEN, KAN, STR, CIP, NOR, SXT, CHL	*aac(6′)-Ic*, *bla*_SRT-2_, *tet*(41)	IncFII(Yp)	SRX11631609	JAIHMR000000000.1	GCA_019733035.1
A11	5,390,897	256	51,946	33	87.42	59	<640	R: PEN, AMP, AMC, ERY, TET; S: FEP, CTX, CFM, DOR, IPM, MEM, GEN, KAN, STR, CIP, NOR, SXT, CHL	*aac(6′)-Ic*, *bla*_SRT-2_, *tet*(41)	IncFII(Yp)	SRX11631624	JAIHMT000000000.1	GCA_019733005.1
A14	5,354,901	304	41,773	41	63.73	59.89	<640	R: PEN, AMP, AMC, ERY, TET; S: FEP, CTX, CFM, DOR, IPM, MEM, GEN, KAN, STR, CIP, NOR, SXT, CHL	*aac(6′)-Ic*, *bla*_SRT-2_, *tet*(41)	IncFII(Yp)	SRX11631616	JAIHMW000000000.1	GCA_019732825.1
A16	5,339,838	326	32,860	48	68.91	59.7	<640	R: PEN, AMP, AMC, ERY, TET; S: FEP, CTX, CFM, DOR, IPM, MEM, GEN, KAN, STR, CIP, NOR, SXT, CHL	*aac(6′)-Ic*, *bla*_SRT-2_, *tet*(41)	IncFII(Yp)	SRX11631622	JAIHMY000000000.1	GCA_019732795.1
A17	5,383,868	267	42,832	37	90.24	59.2	<640	R: PEN, AMP, AMC, ERY, TET; S: FEP, CTX, CFM, DOR, IPM, MEM, GEN, KAN, STR, CIP, NOR, SXT, CHL	*aac(6′)-Ic*, *bla*_SRT-2_, *tet*(41)	IncFII(Yp)	SRX11631615	JAIHMZ000000000.1	GCA_019732715.1

a The wastewater treatment plants service a city that has a population of more than 22,000 individuals and is located in the Atlanta, Georgia, metropolitan area.

b The MIC of colistin was determined using the broth microdilution assay described in the Clinical and Laboratory Standards Institute (CLSI) guidelines (18).

c Resistance to antibiotics was determined using the disk diffusion assay and the CLSI guidelines (18). R, resistance; S, susceptibility; PEN, penicillin; AMP, ampicillin; AMC, amoxicillin-clavulanic acid; FEP, cefepime; CTX, cefotaxime; CFM, cefixime; IPM, imipenem; DOR, doripenem; MEM, meropenem; GEN, gentamicin; KAN, kanamycin; STR, streptomycin; ERY, erythromycin; TET, tetracycline; CIP, ciprofloxacin; NOR, norfloxacin; SXT, trimethoprim-sulfamethoxazole; CHL, chloramphenicol. The antibiotics in the resistance profiles are arranged according to the order of antibiotics/classes listed in the CLSI guidelines.

The draft genome sequences shed light on the resistance of circulating *Serratia* spp. and the role of sewage as a sink for antibiotic-resistant bacteria and resistance determinants.

Ethics approval was not required for this study.

### Data availability.

The raw sequences and the assembled genome sequences for the analyzed strains were deposited in GenBank. The accession numbers for all of the sequences are listed in Table 1.
